# Single nucleotide polymorphisms in the growth hormone receptor gene and *Alu1* polymorphisms in the diacylglycerol acyltransferase 1 gene as related to meat production in sheep

**DOI:** 10.14202/vetworld.2020.884-889

**Published:** 2020-05-14

**Authors:** Nada H. Altwaty, Lamiaa M. Salem, Karima F. Mahrous

**Affiliations:** 1 Department of Biological Sciences, Faculty of Science, King Abdulaziz University, Jeddah, Saudi Arabia; 2 Department of Cell Biology, National Research Centre, Dokki, Giza, Egypt

**Keywords:** genetic polymorphism, polymerase chain reaction-restriction fragment length polymorphism, sequencing, sheep

## Abstract

**Aim:**

This study aimed to investigate the polymorphisms in genes related to meat production, including growth hormone receptor (*GHR*) and diacylglycerol acyltransferase 1 (*DGAT1*) genes, in different breeds of sheep, including Barki, Najdi, and Harri.

**Materials and Methods:**

Blood samples were collected from 75 randomly selected healthy Barki, Najdi, and Harri breeds of sheep, with 25 samples per breed. *GHR* and *DGAT1* genes were identified using a single nucleotide polymorphism assay followed by digestion with the restriction enzyme *Alu1*.

**Results:**

The analysis of the *GHR* gene sequence showed nucleotide substitutions at nt 69 in exon 10 (c.69 G > A); this mutation is considered a transition mutation. The sequences of detected SNPs in the *GHR* gene in the different sheep breeds were submitted to the GenBank database with accession numbers MG906773 to MG906781. The substitutions at exon 10 (c.69 G > A) results in an alteration to the amino acid (p. Lysine > Arginine). At c.69, the A allele frequency was 0.61, 0.59, and 0.54, while the G allele frequency was 0.39, 0.41, and 0.46, for Barki, Najdi, and Harri breeds, respectively. The genotype AG at nt 69 locus had the highest frequency in the Najdi and Harri sheep. The frequency of AG was 0.62, 0.61, and 0.64, while the frequency of AA was 0.30, 0.28, and 0.22, for Barki, Najdi, and Harri sheep, respectively. After digestion with the restriction enzyme *AluI*, the *DGAT1* locus had two genotypes, CC and CT. The highest frequency, 0.88, was found for allele C, which was detected in Barki breed. The lowest frequency, 0.75, for the same allele was found for Harri.

**Conclusions:**

The detected CT genotype may explain the moderate intramuscular fat content and muscle marbling in the Barki sheep breed.

## Introduction

Goats and sheep are two of the most ancient domesticated farm animals, arising over 10,000 years ago [1]. Sheep and goats are among the most economically beneficial farm animals, as they consume cheap low-quality feed and are able to survive in unfavorable climates. Reports demonstrate how sheep can thrive in a variety of environments, including extreme environments ranging between very cold and very hot conditions, as well as in flat and mountainous regions [2]. Sheep provide protein in the form milk and meat, and reports suggest that meat from sheep represents about 4% of the total amount of meat eaten globally [2]. The consumption of meat is increasing gradually and lamb is an important part of this healthy diet [3]. However, red meat producers worldwide are unable to meet the demands for red meat [3]. In addition to meat and milk, sheep also provide important textile materials, such as thread and wool, which are important sources of animal fiber. In undeveloped countries, sheep also provide fertilizer, pelts, and organic fuel [4].

Studying the variation in genes that affect sheep breeding is very important to differentiate between sheep breeds based on their genetics [5]. In addition, genetic study of variation in sheep genes allows the application of the best improvement strategies in sheep breeding, which may depend on the knowing the genotype of these candidate genes [5]. The growth hormone receptor gene (*GHR*) belongs to a super-family of cytokine receptors (i.e., hematopoietin receptors). The *GHR* gene encodes a protein which contains a large cytoplasmic domain, a binding site for extracellular hormones, and a single transmembrane domain that is 24 amino acids long [6]. Although it is known that the GHR plays an essential role in regulation of growth, there is little knowledge about the role polymorphisms in the *GHR* gene play in ovine and bovine production, including growth and meat levels [7-10]. In the most species of mammals, *GHR* gene contains 50 untranslated regions and nine translated exons [11]. Several studies have reported a correlation between the variation in the non-coding region of the *GHR* gene and production of meat in bovines [12,13].

*DGAT1* encodes an enzyme that is involved in triglyceride synthesis and plays a key role in glycerolipid metabolism in adipocytes [14]. Therefore, DGAT1 is involved in the regulation of fat deposition and synthesis in mammals [15]. Several reports indicate that upregulation of DGAT1 enhances triglyceride storage in the cells of multiple tissues, including skeletal muscle, liver, and adipose tissues. Upregulation of DGAT1 leads to low levels of free fatty acids and diacylglycerol [16,17]. Increased levels of fatty acids and diacylglycerol are associated with lipotoxicity, increased inflammation, and cell death [18]. Therefore, higher expression of DGAT1 results in the suppression of lipotoxicity, through conversion of free fatty acids and diacylglycerol to triglycerides. Upregulation of DGAT1 in adipose cells may increase the development of obesity in animals that are supplied with a high-fat diet [18]. Other work has suggested that increased expression of DGAT1 protects mammalian cells from inflammation and improves the cells response to alterations in insulin levels [19].

The aim of the present work was to detect the genetic polymorphisms of two genes important for meat production, *GHR* and *DGAT1*. Breeding based on the genotypes for these two genes has the potential to modulate body weight in sheep breeds in Egypt and Saudi Arabia.

## Materials and Methods

### Ethical approval and informed consent

Permission for collecting the samples used in this work was received from the management of sheep farms and slaughterhouses belong to National Research Centre and King Abdulaziz University, respectively, that were included in this study. The samples were collected per standard sample collection procedures without any harm to animals.

### Animals and DNA extraction

The blood samples were collected from 75 randomly sampled sheep, including Barki, Najdi, and Harri breeds. Healthy Barki samples (n=25) were collected from animal station farm, Faculty of Agriculture, Cairo University, Egypt, whereas Najdi (n=25) and Harri (n=25) samples were collected from a slaughterhouse in the Kingdom of Saudi Arabia.

Genomic DNA was extracted from the whole blood according to the methods described by Miller *et al*. [20] with minor modifications. DNA concentration and purity were determined using a NanoDrop 1000 Thermo Scientific spectrophotometer. DNA was then diluted to the working concentration of 50 ng for polymerase chain reaction (PCR).

### PCR

The amplification of DNA was performed for the *GHR* and *DGAT1* genes using the PCR technique developed by Mullis *et al*. [21] using the primers listed in Table-1. The amplification reaction consisted of 100 ng DNA, 1.0 M primers, 0.2 mM dNTPs, and 1.25U of Taq polymerase. The PCR reaction was cycled under the following conditions: Initial denaturation for 5 min at 94°C followed by 35 cycles of denaturation at 94°C, annealing at 60°C, and extension at 72°C, at 1 min for each step, and the final extension was at 72°C for 5 min. The amplification was verified by horizontal electrophoresis on a 2% agarose gel using the GeneRuler 100 bp ladder. The gel was stained with ethidium bromide and visualized on a UV transilluminator.

**Table-1:** The identification of the primer sequences.

Gene	Primer sequences	References
*GHR*	GCCAAAACAATAAGACTGGGAACC GGCTGTAGTGGTAAGGCTTTCTGT	[20]
*DGAT1*	GCATGTTCCGCCCTCTGG GTCCTAAATAGGTCCTCTCG	[30]

### Sequencing analysis of *GHR* gene

The PCR products of the *GHR* gene were purified and sequenced by Macrogen Incorporation (Seoul, Korea). Sequence analysis and alignment were carried out using NCBI/BLAST/blastn suite. The nucleotide sequence of the Egyptian and Saudi sheep was submitted to GenBank (NCBI, Banklt).

### Restriction fragment length polymorphism (RFLP) for *DGAT1* gene

The PCR products of *DGAT1* gene were digested using *AluI* FastDigest restriction enzyme (Fermentas) for 5 min at 37°C. The restriction fragments were subjected to electrophoresis on a 2% agarose gel stained with ethidium bromide (GIBCO, BRL, England) in 1X TBE buffer. Gels were visualized under UV light and documented in a FX Molecular Imager apparatus (BIO-RAD, http://www.labimaging.com).

## Results

### SNPs in the *GHR* gene in Barki, Najdi, and Harri sheep

**Figure-1:**
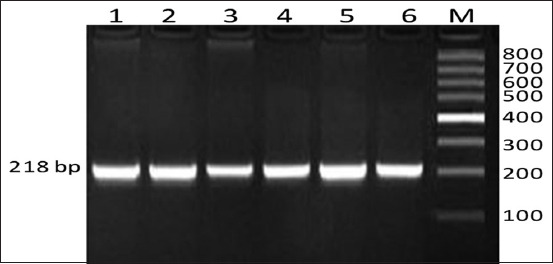
Detection of polymerase chain reaction products of growth hormone receptor gene on 2% agarose gel electrophoresis. Allele size for the growth hormone gene was about 218 bp. Lane M, 100 bp molecular size marker. Lane (1-6) sheep breeds.

**Figure-2:**
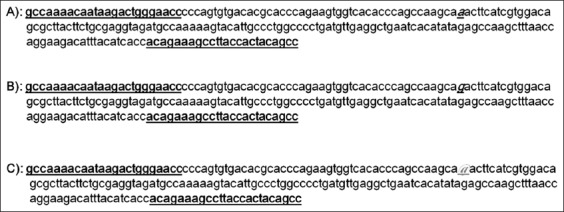
Nucleotide sequence of 218 bp growth hormone receptor amplified fragment in Barki, Najdi, and Harri sheep. Exon 10 is in bold. (A) Allele “a” is detected at nt-69 is in italic and underlined. (B) Allele “g” is detected at nt-69 is in italic and underlined. (C) Allele “@” is a SNP (A/G) at nt-67 is in italic and underlined. Forward and reverse primers are underline.

**Figure-3:**
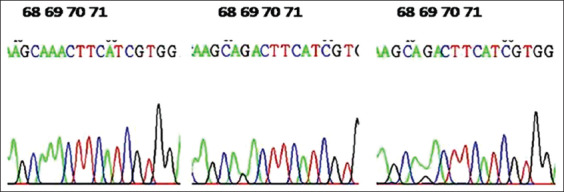
Demonstration of sequenced results; the green blue color indicates the mutation sites.

**Figure-4:**
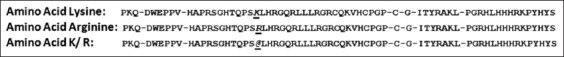
Amino acids sequence alignment of growth hormone receptor exon 10 showed amino acid lysine (K), arginine (R), and @ representing lysine/arginine the heteromorphic variants in three studied breeds.

**Figure-5:**
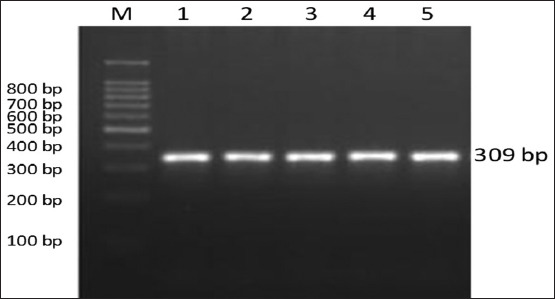
Detection of polymerase chain reaction products of diacylglycerol acyltransferase 1 (*DGAT1*) gene on 2% agarose gel electrophoresis. Allele size for the *DGAT1* was about 309 bp. Lane M: 100 bp DNA ladder. Lanes (1-5) sheep breeds.

The genetic polymorphisms of two genes related to meat production and meat quality, *GHR and DGTA1*, were examined in three sheep breeds, Barki, Najdi, and Harri. The PCR amplified products for *GHR* produced a 218 base pair (bp) fragment in the Egyptian and Saudi sheep (Figure-1), which were expected based on the primers designed for the *GHR* gene, which spanned exon 10 from nucleotide 53 to 270 within exon 10. This PCR amplicon was purified and sequenced for SNP identification. **S**equences were aligned using the GenBank database for detecting the nucleotide differences. Variation in nucleotide sequences between the investigated samples and those of sheep published in GenBank was assessed using NCBI/Blast sequences (accession number AY292283, http://blast.ncbi.nlm.nih.gov/Blast.cgi). There was 99% similarity between with samples, with a difference only at position 69 in exon 10, where there was a SNP (G>A) (Figures-2-5).

The sequence analysis revealed two nucleotide substitutions at nt 69 in the exon 10 (A>G). Accordingly, two bases were observed at these locations, G and A. Three corresponding genotypes were observed: GG, GA, and AA. These genotypes, GG, AG, and AA at nucleotide 69 are shown in Figures-2-5, respectively. The sequences of the detected SNPs in the *GHR* gene in different sheep breeds were submitted to the GenBank database with accession numbers of MG906773 to MG906781. Amino acid sequence alignment was performed on GHR (exon 10) of different sheep breeds showing amino acid lysine (K), arginine (R) at representing lysine/arginine the heteromorphic variants. The substitutions at exon 10 nt 69 (A > G) lead to changes in the amino acid sequence (p. Lysine > Arginine). Amino acid sequences were the same except at this codon, 23, and the alteration at this location was the result of the presence of the A/G variant in the nucleotide sequence. Amino acid variation is presented by lysine (A *A* A)/arginine (A *G*A).

The genotypic frequency for AA was 0.30, 0.28, and 0.22 and genotypic frequencies of AG were 0.62, 0.61, and 0.64 for Barki, Najdi, and Harri, respectively (Table-2). The A allele frequency was 0.61, 0.59, and 0.54, while the G allele frequency was 0.39, 0.41, and 0.46 for Barki, Najdi, and Harri, respectively (Table-2).

**Table-2:** Percentage of genotypic and allelic frequencies of *GHR* gene.

Breeds	Genotypic frequency			Allele frequency	
	
	GG	AG	AA	G	A
Barki	0.08	0.62	0.30	0.39	0.61
Najdi	0.11	0.61	0.28	0.41	0.59
Harri	0.14	0.64	0.22	0.46	0.54

### RFLP in the diacylglycerol acyltransferase1 gene of Barki, Najdi, and Harri sheep

**Figure-6:**
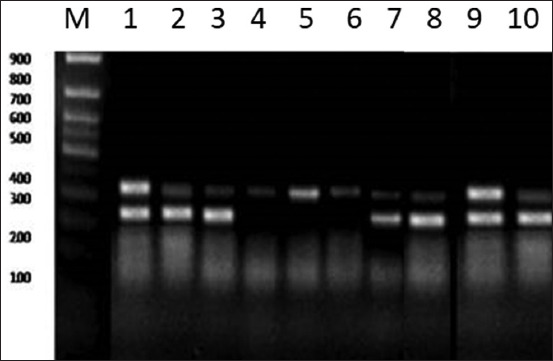
DNA electrophoretic pattern of diacylglycerol acyltransferase 1 amplicon after digestion with *Alu1* endonuclease. Lane M: 100 bp DNA ladder, Lanes genotype CC (4, 5, 6) (309 bp) and Lanes (1, 2, 3, 7, 8, 9, 10) genotype CT (309, 272, and 37 bp).

The PCR amplification of the *ovine DGTA1* gene produced a DNA fragment of 309 bp (Figure-6). The digestion of the PCR amplicon by restriction endonuclease *Alu1* generated two fragments, one with the a C (309 bp) and one with a T (272 and 37 bps). The two genotypes, CC (309 bp) and CT (309, 272, and 37bp), were observed in all breeds. The TT genotype was absent in all breeds (Figure-6). The genotypic frequency of CC was 0.75, 0.65, and 0.50 and genotypic frequency CT was 0.22, 0.35, and 0.50 for Barki, Najdi, and Harri, respectively (Table-2). The C frequency was 0.89, 0.83, and 0.75, while the T frequency was 0.11, 0.17, and 0.25 for Barki, Najdi, and Harri, respectively (Table-2). Heterozygotes showed different mean values, 0.25 for Barki, 0.40 for Najdi, and 0.50 for the Harri breed; however, the mean values of heterozygosity were expected to be 0.25 for Barki, 0.33 for Najdi, and 0.40 for Harri, respectively (Table-2). The *DGTA1* locus *Alu1* had a Chi-square value of 1.96, 1.47, and 0.00 for Barki, Najdi, and Harri (Table-3), respectively.

**Table-3:** Genotypic, allelic frequencies, observed heterozygosity, expected heterozygosity and χ^2^ estimates of *DGTA1* gene digested with *Alu1.*

Breeds	Genotypic Frequency			Allelic frequency		Ho	He	χ^2^
	
	CC	CT	TT	C	T			
Barki	0.78	0.22	0.00	0.89	0.11	0.25	0.25	1.92
Najdi	0.65	0.35	0.00	0.83	0.17	0.40	0.33	1.43
Harri	0.50	0.50	0.00	0.75	0.25	0.50	0.40	0.00

## Discussion

The *GHR* gene was assessed as a prospective candidate gene for a QTL-influenced meat production trait. Exon 10 of the *ovine* GHR gene was amplified and produced a 218 bp fragment [22]. The sequence analysis in the present work revealed two nucleotide substitutions at nt 69 in exon 10 (SNP position) and a non-synonymous substitution was observed at the two positions G>A (transition). This led to an alteration in the amino acid sequence (p. Lysine > Arginine) of the GHR. Three different patterns (GG, GA, and AA) were found at the SNP position at nt 69 in the three sheep breeds studied. With regard to allelic frequencies, the G nucleotide base was predominant in all breeds studied. The highest frequency for A allele was found in Harri while the lowest one was observed in Barki.

Relatively similar results were obtained in another study by Valeh *et al*. [22] performed on Iranian sheep (Baluchi breed) using single strand conformational polymorphism (SSCP). They found two alleles (G and A) in the Baluchi breed which produced the GG, AG, and AA genotypes with frequencies of 7.8%, 61%, and 31.2%, respectively. Other evidence alsoindicates that several cattle breed’s exhibit genetic variation in the *GHR*/*Alu*I site [23]. This study reported that exon 10 has a unique SNP in the coding region of the cytoplasmic domain of *GHR* (c.81 A > G) that leads to an amino acid substitution (Serine > Glycine).

The results are in a close agreement with those obtained by Yuan *et al*. [13], who found a unique SNP in the *GHR* gene, (c.300 A >G) accession number: AY643087. Another study by Hadi *et al*. found a unique SNP in the *GHR* gene (c.149 A > G) accession number: AF126288 [24]. Maj and Zwierzchowski investigated the impact of the *GHR* gene on characteristics associated with meat production and feed consumption in cattle [25]. This study identified a specific domain in the 5’-non-coding region of the *GHR* gene which is involved in meat production in cattle. In contrast, there is a study that investigated the diversity of position 257 on exon 10 and different bovine meat production phenotypes corresponding with the genetic diversity [8]. This study found no correlation between meat production characteristics, including size, growth, and meat conformation, based on nucleotide substitutions on the *GHR* gene at this locus. However, they found a significant correlation between drip losses at day 3 of the meat character and nucleotide substitution (G>A) in the *GHR* gene (A over G) and higher meat value. Several studies have reported that there are various SNPs in exon 10 of the *GHR* gene of sheep (3 SNPs) and cattle (14 SNPs) [26-28]. El-Magd *et al*. [29] reported a correlation between variants, including SNP variants, in the *IGF-1* gene, and bovine growth traits. Yilmaz *et al*. [30] reported a correlation between allele variants in sheep breeds and growth traits.

The amplified segment of the *DGTA1* amplicon includes part of exon 16, whole intron 16, and exon 17. Xu *et al*. [31] identified a nucleotide substitution C> T on exon 17, which leads to a new cut site (AGCT) with the restriction enzyme *Alu1*. If there is a C at this position the band produced will be 309 bp, while if there is a T at this position the bands will be 272 and 37 bp. Two genotypes were generated after digestion with *Alu1*, CC (309 bp) and CT (309, 272, and 37 bp), while the third genotype TT was absent in the present study. Yang *et al*. demonstrated that this SNP is a silent substitution on the sequences encoding alanine (GCC over GCT), which made no substitution alteration for the sequence of the amino acid of DGTA1 protein [32].

The genotypic and allelic frequencies of the *DGTA1* gene in exon 17 of the studied breeds showed that Barki and Najdi breeds have the CC genotype with the higher frequency than the CT genotype, while in the Harri breed, the two genotypes CC and CT were similar in frequency. The TT genotype was not found in any of the three breeds studied. With regard to allelic frequencies, the C base was the predominant one in all breeds. The highest frequency for the T allele was found in Harri while the lowest one was seen in Barki. These results are in agreement with those previously reported, which suggested that the CC genotype was more frequent than the CT and TT genotypes in Tan sheep, Oula sheep, Ganjia sheep, and Qiaoke sheep [32]. In addition, the SNP (C>T) in exon 17 of Chinese indigenous sheep breeds had a significant association with intramuscular fat content (IMF), muscle marbling, and meat tenderness. The Chi-squared values in the present study were within HWE (p>0.05) which is similar to the previous findings, where the allele distribution in Tan sheep and Oula sheep was in Hardy–Weinberg equilibrium [32]. Xu *et al*. [33] reported that the substitution of T>A in *DGTA1* affects the meat production and quality traits of sheep with the CT having a moderate IMF content and marbling score. Detection of the CT genotype and absence of the TT genotype in the studied breeds may explain the moderate IMF content and muscle marbling in the sheep breeds.

## Conclusion

The sequence analysis revealed two nucleotide substitutions at nt 69 in the exon 10 (c.69 A>G) (SNP position). Accordingly, three genotypes were observed GG, GA, and AA. The substitution (c.69 G>A) is a new variant (p. Lysine>Arginine). Harri sheep showed the highest genotypic frequency (CT) and the highest observed heterozygosity for the *DGTA1* gene, followed by the Najdi and Barki sheep.

## Authors’ Contributions

All authors participated equally in the study plan and design. NHA, LMS, and KFM collected the samples from different locations and isolated the nucleic acids. LMS and KFM carried out PCR and sequencing analyses. NHA and KFM carried out the statistical analysis of data and reported the results of the molecular analysis. NHA, LMS, and KFM collaborated on writing, revising, and improvement of the article for publication. All authors read and approved the final manuscript.
